# ADHD symptoms and school impairment history in parents of ADHD children are a fundamental diagnostic and therapeutic clue

**DOI:** 10.1186/s13052-022-01240-7

**Published:** 2022-03-28

**Authors:** Luisa Cortellazzo Wiel, Francesco Rispoli, Giulia Peccolo, Valentina Rosolen, Egidio Barbi, Aldo Skabar

**Affiliations:** 1 University of Trieste, Trieste, Italy; 2 Institute for Maternal and Child Health – IRCCS “Burlo Garofolo”, Trieste, Italy

**Keywords:** Attention Deficit Hyperactivity Disorder, Familiarity, Wender Utah Rating Scale-25, School impairment, Emotional lability

## Abstract

**Background:**

Attention Deficit and Hyperactivity Disorder (ADHD) is a multi-factorial condition, with inheritance playing a major role. Recognizing parents’ ADHD represents a clue not only for an earlier diagnosis of the disease in their children, but also to optimize psycho-educational therapy outcomes, by addressing the impairment of parenting related to untreated ADHD. This study aimed to assess the frequency of features suggestive of ADHD during childhood among parents of affected children, and the presence of school and emotional impairment.

**Methods:**

We administered the Wender Utah Rating Scale-25, a self-assessment tool for the retrospective identification of symptoms consistent with ADHD during childhood, to a cohort of 120 parents of 60 children with ADHD, and to a consistent number of “controls”.

**Results:**

The WURS-25 proved positive in 49.1% of fathers and 30.0% of mothers of ADHD patients, compared to 1.7% of fathers and 1.7% of mothers of non-ADHD patients (*p* < 0.0001).

The questions addressing learning and emotional impairment provided significantly higher scores in parents with an overall positive test compared to those with negative test (*p* < 0.0001).

**Conclusions:**

This study demonstrates a remarkably high rate of symptoms consistent with ADHD during childhood in parents of affected children. Physicians should be aware that this is a relevant anamnestic clue and, given the relevance of parents’ role in the management of children with ADHD, an important issue to address in order to optimize patients’ treatment.

## Background

Attention Deficit and Hyperactivity Disorder (ADHD) is a condition characterized by marked, persistent, maladaptive levels of inattention, impulsiveness, and hyperactivity, which has a negative impact on social, educational, and professional performances. Its estimated prevalence worldwide is 5% in children and 2.5% in adults [1], with studies showing that in half the cases, the disorder persists during adulthood [2, 3]. According to a survey [4], 40% of children diagnosed with ADHD undergo remission during adulthood, in 40% of cases symptoms persist in an attenuated form with related emotional deregulation, social and professional difficulties, while 20% continue to show features consistent with the typical form of the disease.

Although not yet fully understood, ADHD aetiology is probably multi-factorial. Several factors have been demonstrated to be involved, including low birth weight, smoking, drug and alcohol exposure during pregnancy, among biological factors [5], and maternal psychiatric disorders, family dysfunction and lower socioeconomic status among environmental modifiers [6]. The identification of specific causative genes is still hampered by the significant phenotypic heterogeneity of the disorder [7]. Nevertheless, the role of inheritance has been well clarified [8]: twins concordance amounts to 70–76% [9–11] and the presence of an affected first-degree relative has been demonstrated to give to any child a four times higher risk to develop the disorder [12].

This study aimed to assess the frequency of features suggestive of ADHD during childhood among parents of affected children, and the presence of school difficulties and emotional lability.

## Methods

A prospective case–control study was performed at the Child Neurology and Psychiatry Unit of the University teaching, tertiary children’s hospital, Institute for Maternal and Child Health “Burlo Garofolo”, in Trieste, Italy, from April 2019 to October 2019. The study was approved by the Institutional Review Board and all participants gave their written consent to take part in it.

**Fig. 1:**
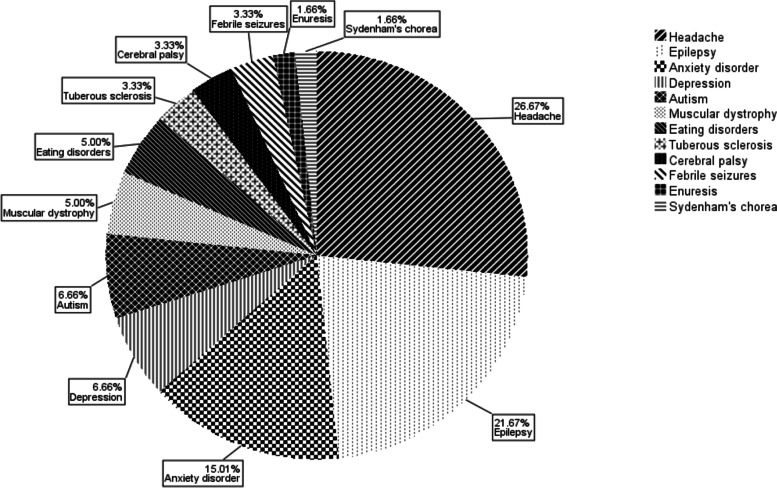
Co-morbidities in non-ADHD patients

We considered as “cases” the parents of children who had received a diagnosis of ADHD (of any of the following types: impulsive/hyperactive, inattentive and distractible, combined) between January 2005 and June 2019. The control group consisted of parents of children with various neuropsychiatric conditions, summarized in Fig. 1, and did not include those of patients who presented other conditions leading to attention or behavioural deficits, potentially acting as confounding factors. Furthermore, since all ADHD patients had an intellective quotient (IQ) over 70, we selected comparable controls, thus excluding also parents of children with IQ ≤ 70.

**Fig. 2:**
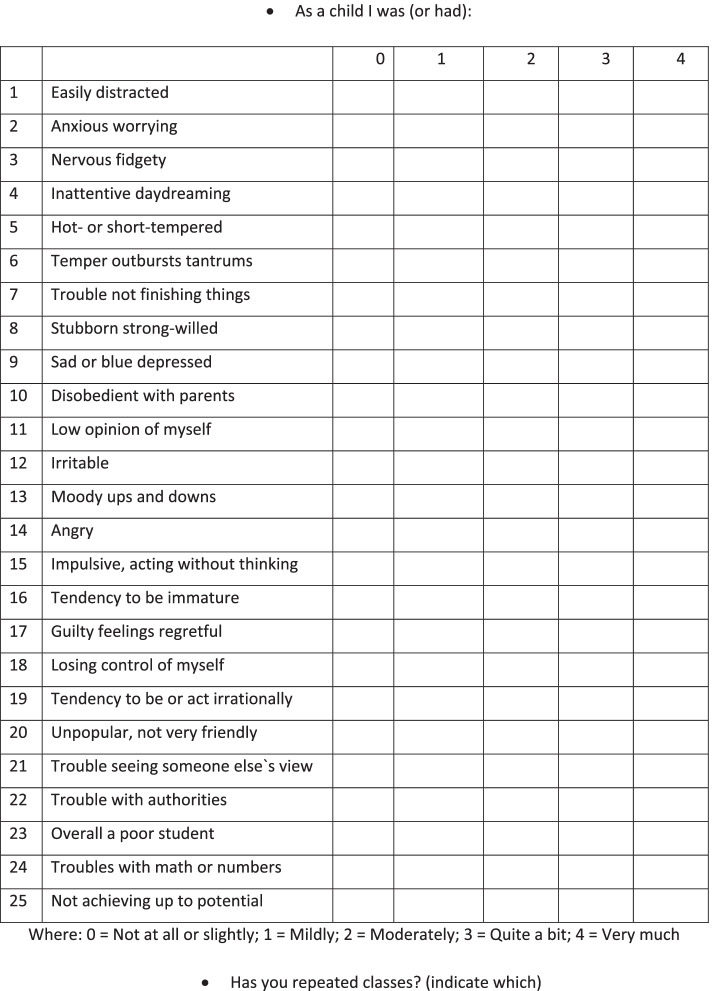
The Wender Utah Rating Scale (WURS) 25

The Wender Utah Rating Scale-25 (WURS-25) [13, 14] in the Italian language (Fig. 2) was administered online to all participating parents. The WURS-25 is a self-assessment tool for the retrospective identification of the presence and severity of symptoms consistent with ADHD during childhood (age 6–10 years). It consists of 25 items, of which 21 address ADHD (inattention, hyperactivity, impulsivity, affective, emotional, and functional dysfunction), and 4 serve as control items. This tool has shown good psychometric properties and satisfactory internal and temporal reliability, and it is considered a screening tool for the retrospective assessment of ADHD in childhood. As ADHD symptoms undergo remission through adulthood in a relevant percentage of cases,when assessing the presence of symptoms suggestive of ADHD in the adult population there is a substantial risk of underestimating the real prevalence of the disorder during their childhood: hence, the WURS represented a suitable tool for this study, providing an accurate picture of the period in which the disorder may have been more expressed.

We instructed the subjects to recall their behaviour and mood during primary school (age 6 to 10), rating every item from 0 to 4 (not at all or very slightly (score = 0), mildly (score = 1), moderately (score = 2), quite a bit (score = 3), or very much (score = 4)). The total score ranges from 0 to 100: we considered 46 as the cut-off score suggestive for previous ADHD 13.

Four questions (number 22–25) aimed to evaluate scholastic impairment and provided an overall score ranging from 0 to 16 points. Seven questions (number 2, 3, 9, 11–13, 17) investigated the presence of emotional lability, providing an overall score ranging from 0 to 28 points.

We used the Chi-Square Test for the dichotomous variables and the Fisher exact test in case of frequencies below 5. For continuous variables, we used the Wilcoxon-Mann Whitney test (for all distributions, the Kolmogorov–Smirnov test for normality had a *p*-value < 0.05).

Results with *p*-value ≤ 0.05 were considered statistically significant.

## Results

Two hundred and two patients were considered for participation in the study; 142 were not included due to the presence of exclusion criteria or to the impossibility to contact their families. Sixty ADHD children (56 males) were therefore finally considered, along with 120 parents (60 mothers and 60 fathers): the latter were considered as “cases” and were matched to 60 mothers and 60 fathers of non-ADHD children as a control group.

Regarding the disease subtypes, 2/60 (3.3%) children had predominant hyperactivity, 14/60 (23.3%) predominant inattention, and 44/60 (74.3%) a combined disorder. Forty-four out of 60 (73%) children had at least one co-morbidity: 35/60 (58%) patients displayed Oppositional Defiant Disorder, 11/60 (23%) Specific Learning Disorder, 10/60 (17%) Mood or Anxiety Disorder, and 7/60 (12%) other disorders, such as mixed learning disorder, language disorder, autism, epilepsy or obsessive–compulsive disorder. Seventeen out of 60 (28%) enrolled patients had more than one co-morbidity. Finally, 46/60 (77%) children with ADHD were on ongoing drug therapy with methylphenidate, in 15/60 (25%) in association with an antipsychotic drug, with 1 patient taking risperidone as a single drug. Thirteen out of 60 (22%) children were not receiving any drug therapy.

Among parents of ADHD children, 60/60 (100%) mothers and 57/60 (95%) fathers answered the questionnaire, versus 60/60 (100%) and 60/60 (100%) mothers and fathers of non-ADHD children, respectively.

**Fig. 3:**
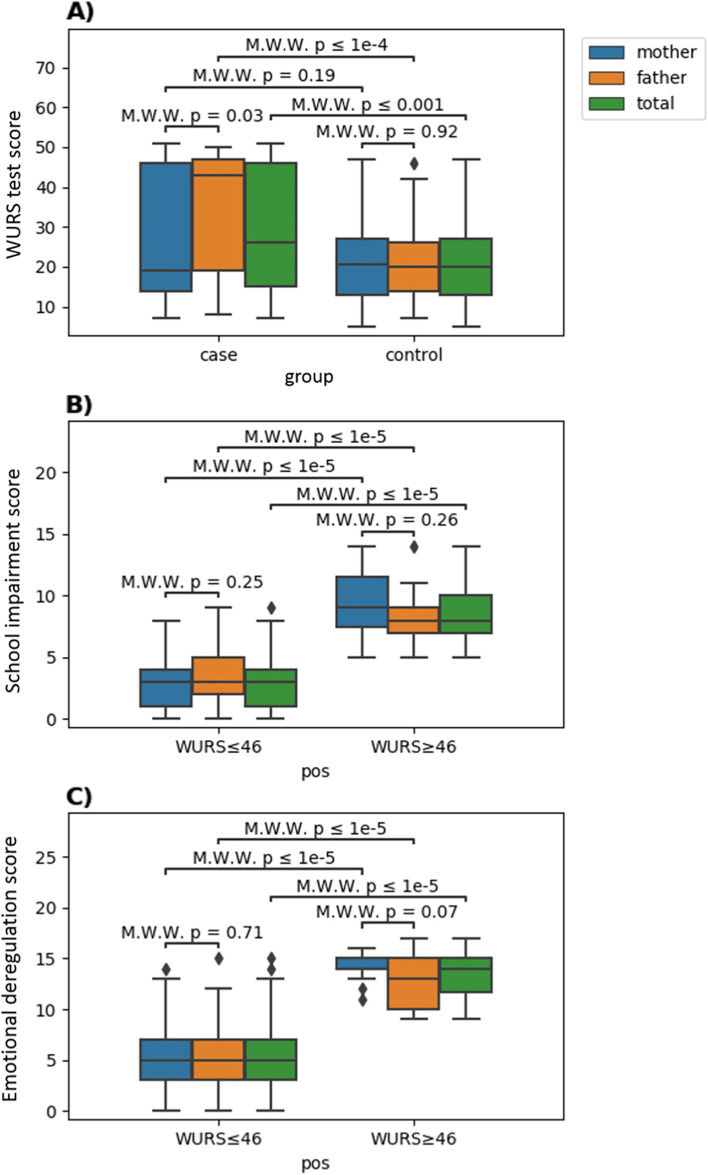
Scores distribution of the WURS-25 test comparing cases and controls, and on questions investigating school impairment and emotional deregulation comparing subjects with an overall positive or negative test

The test proved positive (score ≥ 46) in 46/117 (39.3%) parents of children with ADHD and 2/120 (1.7%) parents of non-ADHD children (χ^2^ = 51.99, *p* < 0.0001) (Fig. 3).

Among parents of ADHD children, we detected a higher rate of positivity in fathers (28/57, 49.1%) compared to mothers (18/60, 30.0%) (χ^2^ = 4.48, p = 0.034), while this difference was not observed among controls (1/60, 1.7% fathers and 1/60, 1.7% mothers; χ^2^ = 0.000, *p* = 1.000).

As for mothers, 94.7% of the positive tests belonged to”the case group”, which meant that they had a child with ADHD (χ^2^ = 17.01, *p* < 0.0001). Similar results were observed in fathers, with a slightly higher correlation (96.6% vs. 3.4%, χ^2^ = 29.43, *p* < 0.0001).

In parents tested positive, a concomitant compromise of school and emotional functioning was sought.

The median score of the questions investigating school impairment was 8 (IQR 7–10) among the 48 parents with overall positive WURS test, versus 3 (IQR 1–4) among the 189 parents testing negative (Table 1, Fig. 3). Of interest, the 28.3% of parents with overall positive WURS test, repeated at least one school year. Through the Wilcoxon-Mann–Whitney test, we compared the school impairment scores of parents with an overall positive and negative WURS test, finding significantly higher scores among parents with a positive test (*p* < 0.0001). The median score was 9 (IQR 7–12) among the 19 mothers who tested positive, versus 3 (IQR 1–4) among the 101 testing negative. A statistically significant difference was found between the two groups (*p* < 0.0001). Besides, 26.3% of mothers with positive test reported having repeated at least one school year. The median score of fathers was 8 (IQR 7–9) among the 29 who tested positive, versus 3 (IQR 2–5) among the 88 testing negative. Even in this case, there was a statistically significant difference between the two groups (*p* < 0.0001), and again, the 27.6% of fathers with positive test reported having repeated at least one school year.

**Table 1:** Parents stratified by study group and WURS-25 test result (positive/negative)

	**Parents of ADHD children**			**Parents of non-ADHD children**			
	**N**	**Negative test**	**Positive test**	**N**	**Negative test**	**Positive test**	***p*-value**
**Total**	117	71 (60.7%)	46 (39.3%)	120	118 (98.3%)	2 (1.7%)	< 0.0001
**Mothers**	60	42 (70%)	18 (30%)	60	59 (98.3%)	1 (1.7%)	< 0.0001
**Fathers**	57	29 (50.9%)	28 (49.1%)	60	59 (98.3%)	1 (1.7%)	< 0.0001

The median score of the questions assessing emotional lability was 14 (IQR 11.5–15) among the 48 parents with positive WURS test, versus 5 (IQR 3–7) among the 189 who tested negative. The difference was statistically significant (*p* < 0.0001) (Table 2, Fig. 3). The median score of mothers was 14 (IQR 14–15) among the 19 who tested positive, versus 5 (IQR 3–7) among the 101 testing negative (*p* < 0.0001). Among fathers, the median score was 13 (IQR 10–15) among the 29 who tested positive, versus 5 (IQR 3–7) among the 88, testing negative (*p* < 0.0001).

**Table 2:** Median and interquartile range (in parenthesis) score of questions assessing school difficulties and emotional functioning stratified by sex with Wilcoxon-Mann–Whitney test *p*-values

		**Positive**		**Negative**		
		**N**	**Median**	**N**	**Median**	***p*-value**
**School difficulties**	**Mothers**	19	9.0 (9)	101	3.0 (8)	< 0.0001
	**Fathers**	29	8.00 (9)	88	3.00 (9)	< 0.0001
	**Total**	48	8.0 (9)	189	3.0 (9)	< 0.0001
**Emotional deregulation**	**Mothers**	19	14.0 (5)	101	5.0 (14)	< 0.0001
	**Fathers**	29	13.0 (8)	88	5.0 (15)	< 0.0001
	**Total**	48	14.0 (8)	189	5.0 (15)	< 0.0001

## Discussion

This study shows a high rate of symptoms consistent with ADHD during childhood in parents of children affected by this disorder.

Several aspects are involved in the multi-factorial pathogenesis of ADHD, including genetic, neurobiological and environmental elements. Inheritance likely plays a fundamental role in pathogenesis: the assessment of its weight would allow not only a better understanding of the disease but also an early diagnosis in the children of affected parents, along with timely treatment and prevention of detrimental consequences. Remarkably, a positive family history may be a relevant diagnostic clue, which physicians should specifically address. On the other hand, parent training is a cornerstone of treatment for children with mild-to-moderate ADHD [15]. Since parental ADHD may particularly impair parenting and family functioning, hindering the ability to deal with affected children, identifying affected parents could be a clue to optimize patients’ outcomes [16, 17].

In this study we found a significant association between kinship and parents’ WURS test positivity: in particular, 49.1% of fathers and 30.0% of mothers of ADHD patients, compared to 1.7% of fathers and 1.7% of mothers of non-ADHD patients, had a positive test, reporting features consistent with ADHD during their childhood. Remarkably none of the surveyed parents had ever been diagnosed with ADHD, underlying a lack of standardized diagnostic criteria to detect this condition in the past. However, the WURS test does not allow the retrospective diagnosis of ADHD: indeed, its aim is to highlight the presence of emotional and behavioural traits consistent with the disorder.

These results are in line with the previous literature. The role of genetic factors has been assessed over time [8] , and it has been confirmed by the high concordance between twins [3, 9, 11]. Bidwell demonstrated a four-time higher risk of developing ADHD in children with affected parents or first-degree relatives, compared to the general population [12]. Previous studies relied on the WURS test to assess symptoms suggestive of ADHD during childhood in parents of affected children. Starck and colleagues found a WURS test positivity in 49.1% of fathers and 27.3% of mothers of ADHD patients [18]: compared to the above mentioned survey, our study was powered by the analysis of data from a control group and by the comparable number of enrolled fathers and mothers, which allowed us to stratify the weight of familiarity by the sex of affected parents. Moreover, to our knowledge, this is the first study that separately examined the school and emotional impairment of parents during their childhood, through a specific sub-analysis of the scores obtained in the specific test questions addressing these issues.

Regarding school performances, we found an association between reported parents’ school difficulties and WURS test positivity, confirming the pivotal role of undiagnosed and untreated ADHD in learning difficulties. The latter, as underlined by Marzocchi and colleagues [7], can be explained in the light of a vicious circle between the deficit of self-regulation cognitive processes and the inability to adopt effective organizational and executive strategies appropriate to the task. This profile negatively affects the performance in the comprehension of written texts, studying, and resolution of arithmetic problems [19, 20]. In this study, we found that mothers with positive tests showed higher scores in the questions investigating school impairment, compared to fathers testing positive. This issue could further demonstrate that in females with ADHD, inattention is usually preponderant compared to hyperactivity, negatively affecting academic performances.

Similar results were found about emotional deregulation. Parents with an overall positive WURS test demonstrated higher scores in the questions assessing emotional lability, compared to parents with negative tests.

Tabassam and Grainger widely described the emotional dysfunction in ADHD children, characterized by sudden emotional changes, dysphoria, irritability, low tolerance to frustration, emotional hyper-reactivity, and emotions-recognition deficits [21]. The concurrent effect of the critical judgment of families, teachers, and peers, easily led to low self-esteem, sense of social rejection and loneliness, which in turn can promote the development of further psychopathologies, such as mood disorders. By stratifying data by gender, we found that the mothers tested positive displayed a higher median score compared to positive testing fathers, underlying the central role of emotional lability within the disorder in females. In a longitudinal study involving 140 children diagnosed with ADHD, Hinshaw found that female subjects had more severe anxiety, depressive symptoms, and more significant difficulties in daily functioning compared to males [22]: at ten years follow-up, girls with ADHD were more likely to display self-injuring behaviours and suicide attempts compared to boys.

This study, not only confirms these data but also highlights a high occurrence of school and emotional impairment in parents of affected children during their childhood, strengthening the suitability of the WURS test for the retrospective assessment of symptoms suggestive of ADHD, considering the crucial role of these two aspects in the diagnosis of the disorder.

The main limits of this study consist of the relatively small sample size and the lack of a correlation analysis between patients and parents, due to the choice to collect and investigate the results in a completely anonymous way. For the same reason, we assessed every parent independently; therefore, it was not possible to compare the association with an affected child with a single positive parent and both parents’ positivity. On the other hand, the guarantee of anonymity provided a parental participation rate of almost 100%, which in turn, represents the strength of this study. A further point of strength was the case–control design, which allowed additional comparative results.

## Conclusions

This study demonstrated a high rate of symptoms consistent with ADHD during childhood in parents of children affected by this disorder. Physicians should actively investigate parents’ history when evaluating children with suspected ADHD. A proper assessment of the parents of these patients would also be crucial to optimize psycho-educational outcomes.

## Data Availability

All data generated or analysed during this study are included in this published article.

## Abbreviations

ADHD: Attention Deficit and Hyperactivity Disorder
IQ: Intellective Quotient
WURS-25: Wender Utah Rating Scale-25
IQR: Interquartile Range
